# An Improved Method for Road Extraction from High-Resolution Remote-Sensing Images that Enhances Boundary Information

**DOI:** 10.3390/s20072064

**Published:** 2020-04-07

**Authors:** Shuai Wang, Hui Yang, Qiangqiang Wu, Zhiteng Zheng, Yanlan Wu, Junli Li

**Affiliations:** 1 School of Resource and Environmental Science, Wuhan University, Wuhan 430079, China; 2010282050194@whu.edu.cn; 2 Institutes of Physical Science and Information Technology, Anhui University, Hefei 230601, China; yanghui@ahu.edu.cn; 3 School of Resources and Environmental Engineering, Anhui University, Hefei 230601, China; x18301096@stu.ahu.edu.cn (Q.W.); wuyanlan@ahu.edu.cn (Y.W.); 4 Anhui Engineering Research Center for Geographical Information Intelligent Technology, Hefei 230601, China; 5 School of Resources and Environment, Anhui Agricultural University, Hefei 230036, China; lijunli866@ahau.edu.cn

**Keywords:** road extraction, deep learning, coordconv, global attention, high resolution

## Abstract

At present, deep-learning methods have been widely used in road extraction from remote-sensing images and have effectively improved the accuracy of road extraction. However, these methods are still affected by the loss of spatial features and the lack of global context information. To solve these problems, we propose a new network for road extraction, the coord-dense-global (CDG) model, built on three parts: a coordconv module by putting coordinate information into feature maps aimed at reducing the loss of spatial information and strengthening road boundaries, an improved dense convolutional network (DenseNet) that could make full use of multiple features through own dense blocks, and a global attention module designed to highlight high-level information and improve category classification by using pooling operation to introduce global information. When tested on a complex road dataset from Massachusetts, USA, CDG achieved clearly superior performance to contemporary networks such as DeepLabV3+, U-net, and D-LinkNet. For example, its mean *IoU* (intersection of the prediction and ground truth regions over their union) and mean *F1* score (evaluation metric for the harmonic mean of the *precision* and *recall* metrics) were 61.90% and 76.10%, respectively, which were 1.19% and 0.95% higher than the results of D-LinkNet (the winner of a road-extraction contest). In addition, CDG was also superior to the other three models in solving the problem of tree occlusion. Finally, in universality research with the Gaofen-2 satellite dataset, the CDG model also performed well at extracting the road network in the test maps of Hefei and Tianjin, China.

## 1. Introduction

The establishment of road databases is of great significance to the development of modern cities. At present, the extraction of road networks from high-resolution remote-sensing images has been widely used in urban services such as urban planning [1], vehicle navigation [2] and geographic information management [3,4]. However, due to the macro-scale nature of remote-sensing images, the complexity of road networks and the interference of other objects (such as parking lots, building roofs and trees), it is difficult to extract roads effectively [5,6].

At present, the methods for remote-sensing artificial road information extraction mainly include pixel-based [7,8,9,10,11,12], object-oriented [13,14,15,16,17,18], and deep learning methods [19,20,21,22,23,24,25,26,27]. Pixel-based methods mainly extract roads by analyzing the spectral information of the pixels, such as road extraction methods based on mean shift [7], adaptive domain [8], threshold filtering and line segment matching rules [12]. Pixel-based road extraction methods mainly use the spectrum of the image, which has a certain effect on simple road network extraction. However, these methods lack information such as the spatial context of the features and the texture structure, which has a “salt and pepper” effect.

In the object-oriented extraction method, the road is taken as an object and the information model is built to extract the road from the remote-sensing images. This method has good noise immunity and applicability. Compared with the pixel method, the accuracy is also improved. For example, Das et al. [13] used the local linear features of multispectral road images combined with the support vector machine model to achieve road extraction. However, Cheng et al. [14] made good use of the texture and geometric features of remote-sensing images in road extraction of rural and suburban areas. In work on urban road extraction, Senthilnath et al. [15], after preprocessing a remote-sensing image, used the method of texture progressive analysis and a normalized cut algorithm to realize the automatic detection of urban roads based on the road structure, spectral information and geometric features. Xin et al. [17] also proposed a road extraction method based on multiscale structural features and a support vector machine and successfully extracted the centerline of the road on two multispectral datasets. Although the extraction performance of such methods has been improved, it is easy to misclassify pixels that are adjacent spatially and have similar structural features. The design of the classification rules is very complicated, and the accuracy of extraction also needs to be improved.

Deep learning methods have been increasingly applied to information extraction from high-resolution satellite images due to their good performance and generalization ability [28,29]. Since Mihi et al. [30] applied deep learning methods to road extraction, other deep learning models have been applied to road extraction research [5,19,20,21,22,23,24,25,26,27]. Because fully convolutional networks (FCN) has achieved good results in semantic segmentation, many excellent FCN-based networks, such as U-net [31], SegNet [32], and DeepLabV3+ [33], have achieved the advanced performance in image segmentation and have been widely used for road extraction. For example, Wei et al. [19] proposed a road extraction model based on the road structure refined convolutional neural network (RSRCNN) and constructed a new loss function that improved road extraction accuracy. Zhang et al. [5] also proposed a new road extraction method based on U-net, which improved the model’s semantic segmentation ability, reduced the loss of information, and effectively improved the accuracy of road extraction. To reduce misclassification in road classification tasks, Panboonyuen et al. [22] proposed an improved deep convolutional neural network (DCNN) framework that introduced the three mechanisms of landscape metrics (LMs), conditional random fields (CRFs), and exponential linear unit (ELU) in the network, which improved the extraction accuracy of the road and reduced misclassification. However, FCN mainly expands the receptive field and obtains context information through continuous pooling layers, which will cause the loss of small targets and important spatial details during the downsampling process. To overcome the loss of spatial information and obtain more global context information, in the 2018 CVPR (Computer Vision and Pattern Recognition) DeepGlobe Road Extraction Challenge, Zhou et al. [23] used dilated convolution to expand the receptive field and save a certain amount of spatial information while acquiring highly discriminative feature information, which improved the accuracy of the extraction and ultimately achieved excellent results in the competition. However, continuous dilated convolution will still cause the loss of spatial information, produce a “chessboard effect” and consume considerable computer memory.

Then, to reduce the loss of spatial information at multiple levels, Liu et al. [34] proposed an important coordinate convolution module. Coordinate convolution adds two channels to the original convolution to store horizontal and vertical pixel information to obtain spatial information. Yao et al. [35] applied coordinate convolution to land-use classification, effectively strengthened the edge information, and improved the accuracy of classification.

Therefore, to reduce the loss of spatial information at multiple levels, strengthen the global context of FCNs and improve the accuracy of road extraction, in this paper we design a novel encoder-decoder network called the coord-dense-global network to extract roads from remote sensing imagery and achieve better performance. DenseNet uses dense connectivity to connect multilevel feature maps and is considered a powerful feature extractor that can effectively reduce information loss [36]; this is very suitable for extending FCN networks for semantic segmentation [37]. The coord-dense-global network mainly use DenseNets to extract multilevel feature maps. The coordconv module is used to strengthen boundary information and fine details. At the same time, the global attention module is also introduced to highlight high-level features in the encoder part, which can maintain the continuity of roads.

The remainder of this paper is organized as follows: Section 2 presents the proposed methods. Section 3 shows the experimental details and results. Section 4 presents the comparison and analysis of the proposed model, and the conclusions are presented in Section 5.

## 2. Materials and Methods

### 2.1. Proposed Network Architecture

In general, convolutional networks are composed of a series of connected convolutional layers in which *L* layers will produce *L* connections. There exists a nonlinear transformation function *F_L_* in each layer, which usually contains convolutions, rectified linear units [38], and pooling. If the input and output of layer *L* are *X_L_*_−1_ and *X_L_*, respectively, the layer transition can be defined as:

(1) $$X_{L}=F_{L}(X_{L-1})$$

This simple layer transition will lead to information loss and weaken the information flow between the layers. DenseNet has been widely used to solve such problems in image segmentation [39], as its dense connectivity allows the reuse of information from previous layers and reduces the number of parameters, making the network more easily trainable. In our dense connectivity module, the feature maps of all preceding layers are connected to subsequent layers, so layer *L* receives the feature maps from all previous layers (*X*_0_, *X*_1_, *X*_2_, …, *X_L_*_−1_) as input. The layer transformation can thus be described as:

(2) $$X_{L}=F_{L}(\left[X_{0},X_{1},X_{2},\cdots ,X_{L-1}\right])$$

where [*X*_0_, *X*_1_, X_2_, …, *X_L_*_−1_] is the concatenation of all feature maps from all preceding layers. The non-linear transformation function *F_L_* often consists of three consecutive parts: a batch normalization layer [40], a layer of rectified linear units, and a convolutional layer. In addition, DenseNet is designed to have a growth rate that suppresses the redundancy of feature maps and improves the efficiency of the network.

**Figure 1:**
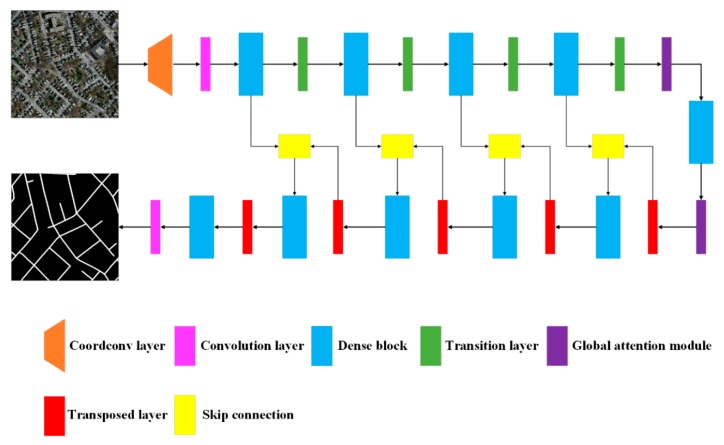
Structure of the proposed coord-dense-global network for improved road extraction from remote-sensing imagery.

Due to the efficiency of dense connectivity, we used an encoder–decoder architecture based on the fully convolutional DenseNet (Figure 1). Because roads are thin and long, we used the coordconv layer to strengthen informational detail and reduce the loss of spatial features in the network’s top layer [35]. In the encoder, a series of dense blocks were applied to extract abstract features, with two global attention layers at the bottom. In the decoder, the high-level features after upsampling were directly concatenated with low-level features from the encoder using skip layers to form a new dense block input. Finally, the binary segmentation map was output following a convolutional layer.

### 2.2. CoordConv Module

**Figure 2:**
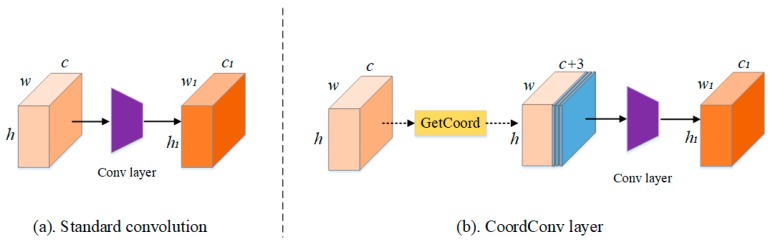
Structural comparison between (**a**) a standard convolutional layer and (**b**) the improved module with the coordconv layer proposed in this study.

The coordconv module is an extension of the standard convolution operation [34] (Figure 2a). Here, coordinate information from the original feature map is extracted and concatenated with original feature maps as input, after which standard convolution is applied (Figure 2b). In general, coordinate information is allocated in the *i* (horizontal) and *j* (vertical) channels. These two coordinates are sufficient to specify input pixels and improve spatial information. Another channel can be used for an *r* coordinate, which is directly calculated from the *i* and *j* coordinates.

(3) $$r=\sqrt{{\left(i-h/2\right)}^{2}+{\left(j-w/2\right)}^{2}}$$

where *h* and *w* are the sizes of the original feature maps.

### 2.3. Global Attention Module

**Figure 3:**
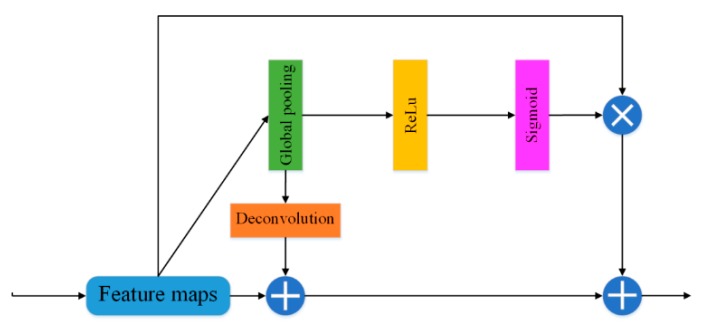
Structure of the global attention module proposed in this study.

As roads are thin, long, and continuous, global context information is important for extraction from remote-sensing images. Because global context information can easily enlarge the receptive field and enhance the consistency of pixelwise classification [41,42,43], we designed a global attention module (GAM) to strengthen high-level features for category classification (Figure 3). The GAM first applies global average pooling to the feature map, obtains the global context vector, and then performs different processing paths. On one path, the deconvolution operation is carried out first, and the results are then added to the original feature map; on the other path, the pooling operation is carried out first. After the activation, the sigmoid classifier is used to normalize the feature vector to [0,1], and then it is multiplied with the original feature map. The terminal of the module adds the output feature maps of the two paths. This module can effectively avoid the loss of global context information and strengthen high-level features while improving segmentation performance.

### 2.4. Implementation

**Figure 4:**
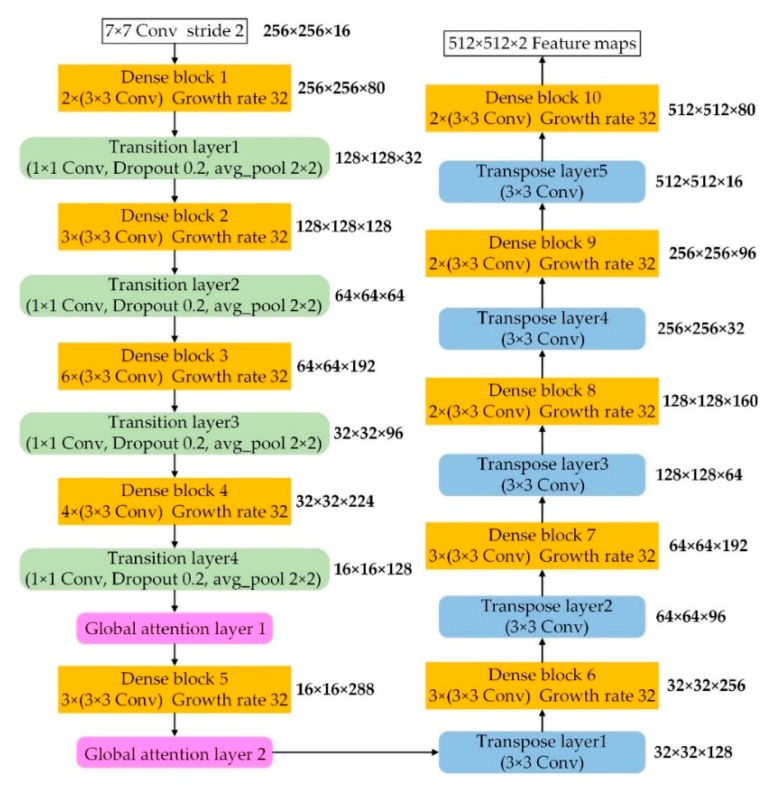
Detailed parameters of the coord-dense-global (CDG) model: orange boxes are dense blocks, green boxes are transition layers, purple boxes are global attention layers, and blue boxes are transition layers.

The concrete parameters of the proposed network are displayed in Figure 4. The growth rate is set as 16, and the convolution operation is 3 × 3 with different numbers of layers in each dense block. For each transition layer, a 1 × 1 convolution operation and a dropout layer with a 0.2 rate are implemented, followed by a 2 × 2 average pooling operation. The transition layer is used to reduce the feature resolution and connect adjacent dense blocks. The transpose layers are opposite, and they recover the resolution. The transpose layer usually adopts a 3 × 3 convolution operation with a 2 stride, and the activation function is often a rectified linear unit (ReLu). After the end of the encoder-decoder process, the feature maps with the same resolution as the input image are output. Due to the binary classification of road extraction, the output is 512 × 512 × 2.

### 2.5. Data

To test the performance of the CDG model, we used a publicly available aerial imagery dataset for roads in Massachusetts, USA [44]. This dataset provided 1171 images with red, green, and blue channels, including 1108 training images, 49 test images, and 14 validation images. All images were 1500 × 1500 pixels with a spatial resolution of 1.2 m. These images cover a wide range of contexts, including urban, suburban, and rural regions, covering an area of 2600 km^2^, and are considered a challenging aerial image labeling dataset [22].

### 2.6. Implementation

We cropped all images to 512 × 512 pixels. Due to the limited number of training samples, we randomly split all labeled images, producing 14,366 subset images for training. We also directly tested the testing data at 1500 × 1500 pixels without other processing. The training used 50 epochs and a batch size of two, according to the graphics processing unit (GPU) memory. To train the network more appropriately, we used the training epochs to automatically adjust the learning rate by dividing the training epochs into several levels and then dividing the learning rate by 10 when the epoch reached the corresponding level. The initial learning rate was 0.001, and the final rate was 0.000001. The total training time was approximately 50 h, and the average testing time of each image at 1500 × 1500 pixels was approximately 1 s. In addition, we used the Adam optimizer [45] to optimize our model and update all parameters because of Adam’s high computational efficiency and low memory requirement.

### 2.7. Evaluation Metrics

We used the *F1* score and *IoU* metrics to evaluate the network’s quantitative performance. The *F1* score is a powerful evaluation metric for the harmonic mean of the *precision* and *recall* metrics, and it is directly calculated as:

(4) $$F1=2\times \frac{precision\times recall}{precision+recall}$$

(5) $$recall=\frac{TP}{TP+FN},\;precision=\frac{TP}{TP+FP}.$$

where *TP*, *FP*, and *FN* represent the number of true positives, false positives, and false negatives, respectively. The *recall* metric represents the number of correct pixels over the ground truth, while the *precision* metric represents the number of correct pixels over the prediction result. These values can be calculated using the pixel-based confusion matrix for each batch.

The *IoU* metric represents the intersection of the prediction and ground truth regions over their union, and the mean *IoU* can be calculated by averaging the *IoU* of all classes:

(6) $$IoU=\frac{TP}{FN+TP+FP}$$

## 3. Experimental Results

**Figure 5:**
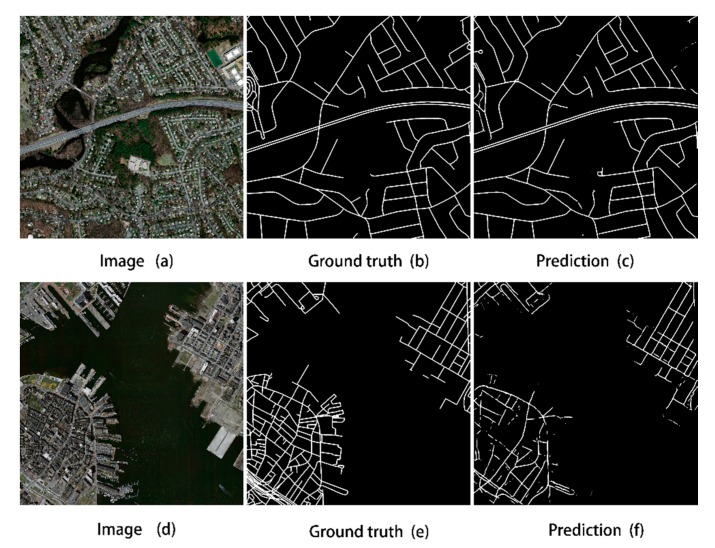
Comparison of the original image (**a**,**d**), ground-truth data (**b**,**e**), and extraction result (**c**,**f**) for the best (**a**–**c**) and worst (**d**–**f**) test cases for CDG.

After training CDG, we evaluated 49 test images, and we saw that the predicted road performance obtained by this method was good, more complete than other networks, and closer to the ground truth image. In order to accurately evaluate the performance, the average of the four evaluation indexes, the *precision*, *recall*, *F1* and *IoU* of all test images are listed in Table 1, which are 81.41%, 71.80%, 76.10% and 61.90%, respectively. At the same time, in order to intuitively understand the extraction effect of the CDG model, we show the best and worst results in the test pictures, as shown in Figure 5.

## 4. Comparison Results and Analysis

### 4.1. Comparison with Other Methods

**Figure 6:**
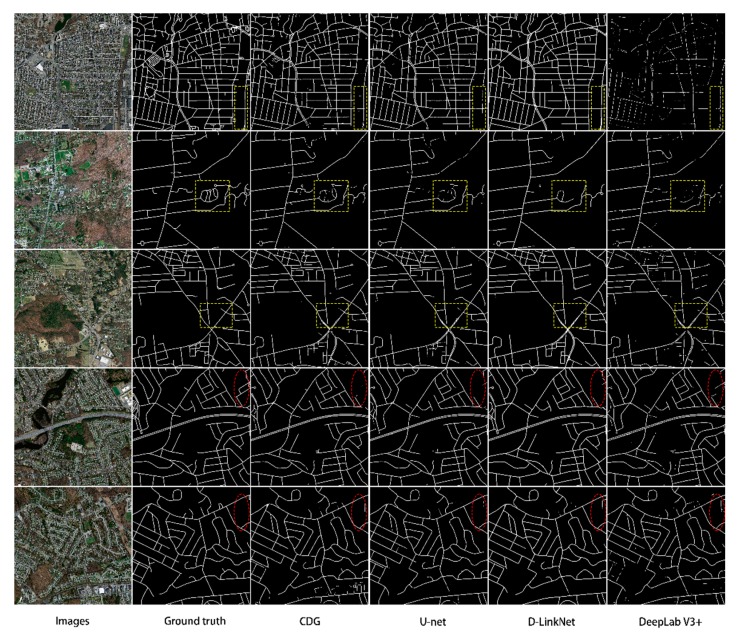
Comparison of results for selected images using CDG, U-net, D-LinkNet, and DeepLabV3+. Yellow boxes highlight areas with differing results between models, while red ellipses highlight areas that consistently had problems in all models.

To assess the relative performance of CDG, we performed road extraction on selected images using three other common methods (DeepLabV3+ [33], D-LinkNet [23], and U-net [31]) and compared the results with our own, as shown in Table 2. As seen from the table, the CDG model is clearly more accurate. Although the CDG model is 2.63% lower than the U-net model with the highest accuracy in the *precision* index, the CDG model has the highest *F1* and mean *IoU* evaluation index, which are 0.95% and 1.19% higher than those of D-LinkNet, respectively. Compared to U-net, they increased by 0.86% and 0.96%, respectively, and far exceeded DeepLabV3+. In addition, in Table 2, we also calculated the *test time* of each model. Although our *test time* is higher than Unet, it has certain advantages in *test time* compared with other models. From a qualitative perspective, we can see that the extraction performance of the method in this paper has improved significantly. Figure 6 shows the road extraction details of the CDG model and the other three models.

From the yellow box in Figure 6, we can clearly see that the extraction results of D-LinkNet and U-net are relatively fragmented, and many small roads in the remote-sensing image also have extraction losses. Among all the extraction results, DeepLabV3+ has the worst extraction effect, and the continuity and integrity of the road are difficult to guarantee. In addition, our analysis found that because the cement structure and road surface are too similar, all the methods have some misclassification problems in this case (see the red ellipses in Figure 6). However, in terms of overall performance, the extraction performance of the CDG model is still optimal.

**Figure 7:**
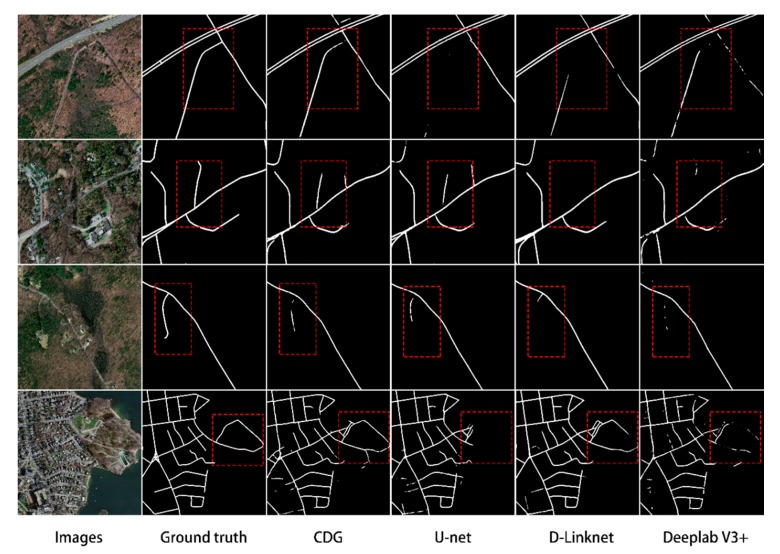
Comparison of results for selected images using CDG, U-net, D-LinkNet, and DeepLabV3+. Red boxes highlight areas with differing results between models.

Figure 7 is a partially enlarged image of the extraction result, which primarily reflects the ability of the CDG model to solve the problem of tree occlusion. It can be seen from the red box in Figure 7 that the CDG model performs better in extracting roads covered by trees and can extract most of the roads covered by trees, while the other three network models perform worse in extracting such roads. Although the D-LinkNet and DeepLabV3+ networks have some ability to extract occluded roads, it is difficult to ensure the continuity of the road, and the result of occluded road extraction is lost. U-net performed the worst in the problem of tree occlusion, and the degree of fragmentation and loss of extraction results was the worst. By comparing and analyzing the three network models DeepLabV3+, D-LinkNet and U-net, it can be found that the CDG network model based on dense links effectively extracts the effective feature information of the road, which improves the integrity and accuracy of the road extraction results. Regarding the coordconv module and the global attention mechanism module, the former can enhance the road edge information and make the road more complete, while the latter can consider global context information and make the road network more systematic in the extraction process. Therefore, the method of this paper can effectively counteract the interference of trees, buildings and other background features and improve the accuracy of road extraction.

### 4.2. Analysis of the Effectiveness of the Mechanism of Action

To highlight the importance of coordinate convolution and the global attention mechanism, this paper compares them with network models lacking coordconv, lacking global attention and lacking both under the same training conditions. In the experiment, we also used 49 test images from the Massachusetts dataset to test the performance of different network models. To quantify the performance of the model, we list the average valuation indicators of all models in Table 3, from which we can see that the CDG model with two mechanisms is far higher than the other three methods in all evaluation indexes. Compared with the no-global-attention model with the highest accuracy, our *precision*, *recall*, *F1* and *IoU* are increased by 2.59%, 2.95%, 2.83% and 4.64%, respectively. It can be seen that the combination of coordinate convolution and the global attention mechanism in the CDG model can effectively reduce the loss of road spatial features, on the one hand, and take into account the global information of the road, on the other hand; it can also enhance the integrity of the road and improve the accuracy of road extraction on the whole.

### 4.3. Generalization Results of the Model

The CDG network model we designed achieved excellent results on the road data set in Massachusetts, USA. To further verify the generalization of the CDG model, we trained and tested it on the Gaofen-2 satellite remote-sensing image. The image resolution is 1 m. In the experiment, we produced 5892 pieces of sample data with a size of 512 × 512. After training, we verified 4 remote-sensing images of size 4578 × 4442 in Hefei, China and Tianjin, China.

**Figure 8:**
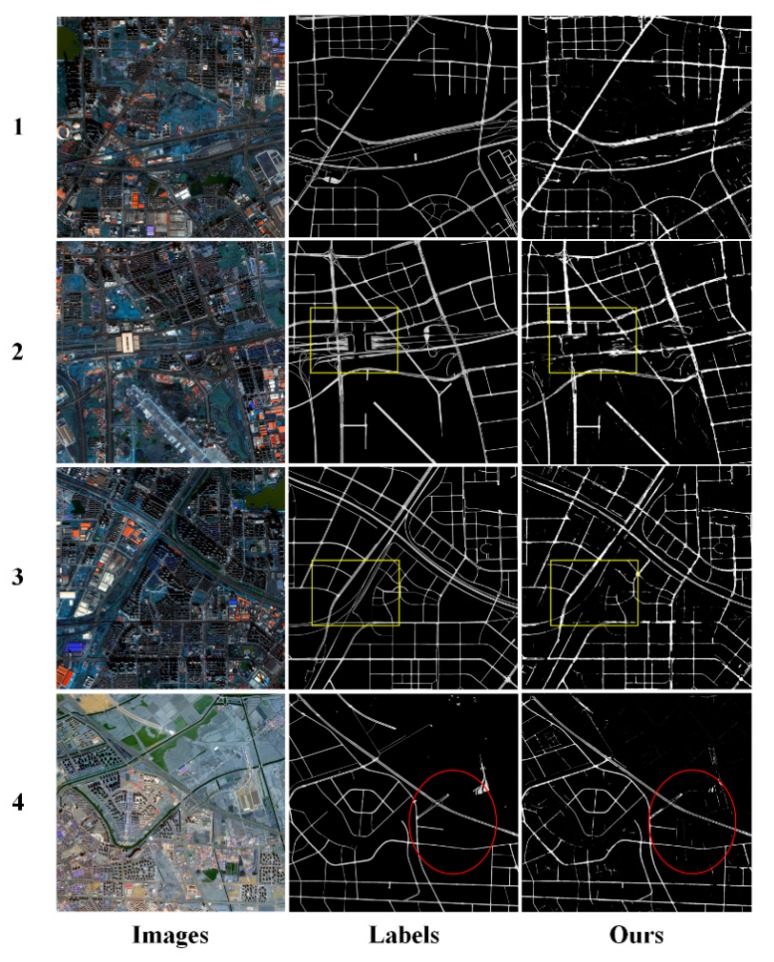
The results of CDG extraction on the Gaofen-2 satellite remote sensing image. Red marker box highlights excellent extraction results, while yellow ellipses highlight areas that consistently had problems.

The test results are shown in Figure 8, we can see that the roads in the Gaofen-2 satellite remote-sensing image were extracted. Although there are missing roads in the image (see the yellow boxes in Figure 8), most of the roads in the image have been extracted; the extraction effect on the main road is particularly excellent, and the degree of agreement with the label is high. At the same time, the CDG model also extracts small roads that are not marked by the label (see the red boxes in Figure 8). To quantitatively evaluate the performance of the CDG model on the Gaofen-2 satellite remote-sensing image, we list various evaluation indicators in Table 4. As seen from the table, the CDG model reached 68.38%, 77.72%, 72.62%, and 57.11% in *precision*, *recall*, *F1* and *IoU*, respectively. This shows that the CDG model is has excellent extraction performance and strong generalization for road extraction tasks.

### 4.4. Problem

**Figure 9:**
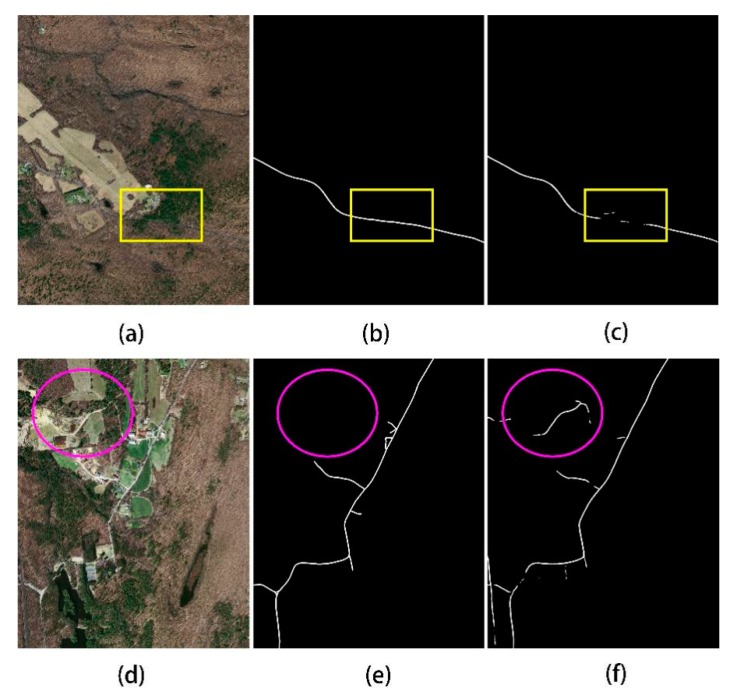
Two examples of problems with CDG model extraction results, comparing the (**a**,**d**) original image, (**b**,**e**) ground-truth data, and (**c**,**f**) extraction results.

Although the CDG achieved improved results, the road is extracted when the road is partially blocked by a low number of trees but, when severely blocked by many trees, the extraction effect of the CDG model is relatively poor. For example, in the previous line of Figure 9, the roads in the ground truth image are continuous (yellow boxes), but discontinuities appear in the predicted image. It may be that the roads are located in rural suburbs, and the trees above the roads are too lush, resulting in low accuracy of the extraction results; many portions of these roads are nearly indistinguishable by eye in the original image. In comparison, the lower row of Figure 9 demonstrates the superiority of the proposed model, where several roads (purple ellipses) were not labelled as roads in the ground-truth image but were successfully identified by the model. Such mismatch between ground truth images and prediction results restricts model performance, but it is difficult to further improve accuracy.

## 5. Conclusions

This paper proposes a deep learning model CDG for road extraction of high-resolution remote-sensing images. The main contribution of this method lies in the introduction of two functional modules based on the Densenet network structure: coordinate convolution and ensemble attention mechanism. The cooperation of the two mechanisms can not only greatly enhance the edge information of road images, but also effectively obtain more global context information. In order to verify the performance of the model, we performed experiments on the M dataset and compared with the D-LinkNet, U-net, and DeepLabV3 + methods. The experiments show that the performance of the CDG model is better than other network models. At the same time, our CDG model is also the best at solving the problem of tree occlusion, and the integrity and continuity of the extraction results are significantly better than other network models. In the last universal experiment in this paper, the CDG model also successfully extracted the roads of Tianjin, China and Hefei, China, and achieved good results. Therefore, CDG is an excellent road extraction model. However, due to interference from complex backgrounds (forests, buildings, different road types and widths, etc.), the extraction results still show discontinuities. In future research, we will consider the use of road geometric information to eliminate incomplete or broken issues in the road extraction process.

## Figures and Tables

**Table 1:** Statistical accuracy assessments for the testing images using CDG.

Testing Dataset	*precision*	*recall*	*F1*	*IoU*
All images-average	81.41%	71.80%	76.10%	61.90%

**Table 2:** Mean accuracy comparisons for four road extraction methods.

Method	*precision*	*recall*	*F1*	*IoU*	*test time*
DeepLabV3+	79.16%	60.22%	67.64%	51.95%	245s
D-LinkNet	79.45%	71.96%	75.15%	60.71%	206s
U-net	84.04%	68.90%	75.24%	60.94%	167s
CDG	81.41%	71.80%	76.10%	61.90%	196s

**Table 3:** Mean accuracy comparisons for four road extraction methods.

Method	*precision*	*recall*	*F1*	*IoU*
no coordconv	76.12%	60.25%	65.75%	49.81%
no global attention	76.20%	75.30%	75.33%	60.96%
neither	81.40%	57.61%	66.76%	50.85%
CDG	81.63%	72.07%	75.94%	61.61%

**Table 4:** Gaofen-2 satellite image extraction accuracy.

Num.	*precision*	*recall*	*F1*	*IoU*
1	62.12%	77.73%	69.00%	52.73%
2	70.88%	75.38%	72.54%	56.97%
3	71.02%	76.93%	73.81%	58.55%
4	69.49%	81.83%	75.11%	60.20%
Average Value	68.38%	77.72%	72.62%	57.11%
